# CircRNA MALAT1/miR-96-5p/FOXK2 axis regulates choroidal neovascularization

**DOI:** 10.1016/j.clinsp.2025.100759

**Published:** 2025-09-24

**Authors:** Yue YIN, Tong Zhao

**Affiliations:** Department of Ophthalmology, China-Japan Friendship Hospital, Beijing City, China

**Keywords:** Choroidal Neovascularization, Age-Related Macular Degeneration, CircRNA MALAT1, miR-96-5p

## Abstract

**Objective:**

This study determined the role of circRNA MALAT1 (circ-MALAT1) in Choroidal Neovascularization (CNV).

**Methods:**

CNV mice were induced by laser photocoagulation, and Hypoxic human Retinal Microvascular Endothelial Cells (HRMECs) were induced using cobalt chloride. circ-MALAT1 expression pattern was determined in CNV mice and hypoxic HRMECs. Hematoxylin-eosin staining and fluorescein fundus angiography were performed to examine focal areas in CNV mice. Endothelial cell proliferation and migration assay, tubule formation assay, and apoptosis assay were conducted to determine angiogenesis *in vitro*. Circ-MALAT1-mediated CNV development was investigated using bioinformatics, luciferase analysis, and RNA immunoprecipitation.

**Results:**

circ-MALAT1 expression was upregulated in laser-induced CNV mice and hypoxic HRMECs. circ-MALAT1 silencing delayed structural disorganization of choroidal membranes and the formation of CNV, which was abolished by miR-96–5p inhibitors. circ-MALAT1 silencing decreased HRMEC viability, proliferation, migration, and tube formation and promoted apoptosis. circ-MALAT1 acted as a miR-96–5p sponge, chelating and inhibiting miR-96–5p activity, which led to increased expression of FOXK2. miR-96–5p overexpression mimicked the anti-angiogenic effects mediated by circ-MALAT1 silencing. FOXK2 overexpression diminished circ-MALAT1 silencing-mediated inhibition of endothelial cell progression. Furthermore, significant upregulation of circ-MALAT1 and FOXK2 expression was detected in clinical samples from neovascular age-related macular degeneration patients.

**Conclusion:**

The introduction of circ-MALAT1/miR-96–5p/FOXK2 as a potential therapeutic target in this study provides new insights into the molecular pathogenesis of CNV, but our preliminary study only hints at the possibility of this axis as a target for CNV intervention, and its clinical significance still needs to be validated with larger sample sizes.

## Highlights

• circ-MALAT1 is upregulated and functions as a pro-angiogenic regulator in laser-induced CNV and hypoxic HRMECs.
• circ-MALAT1 knockdown attenuates CNV lesions in a mouse model.
• circ-MALAT1 knockdown impairs pro-angiogenic functions in HRMECs.

## Introduction

Older adults are commonly affected by Age-Related Macular Degeneration (AMD), a chronic eye disease that causes vision impairment.^1^ The number of AMD patients worldwide is expected to increase to 288 million by 2040. A majority of vision loss is caused by neovascular AMD (nAMD), which is pathologically manifested as Choroidal Neovascularization (CNV).^2^ CNV is characterized by the abnormal growth of choroidal blood vessels, which destroys the bruch membrane and the retinal pigment epithelium layer or hides underneath the retina, and ultimately leads to central vision loss.^3^ Currently, CNV is treated in a variety of ways, but intravitreal injection of anti-Vascular Endothelial Growth Factor (VEGF) has become the gold standard of treatment.^4^ However, frequent injections of anti-VEGF biologics may lead to adverse events such as endophthalmitis and fibrosis. Therefore, an in-depth study of CNV is needed to find new pathogenic mechanisms.

CircRNAs are a class of widely expressed non-coding RNAs with a specific ring structure.^5^ circRNAs are conserved and have roles as functional molecules in physiological and pathological states. Typically, they function by acting as microRNA (miRNA) sponges or interacting with RNA-binding proteins and transcription factors. There are 289 circRNAs with aberrant expression in diabetic retina, and the host genes of these circRNAs are associated with biological processes such as ATP binding, extracellular exocytosis and intracellular signaling.^6^ These circRNAs are enriched in extracellular matrix-receptor interaction and adhesion signaling pathways, suggesting their potential value in the early diagnosis of diabetic retinopathy.^7^^,^^8^ Additionally, circUxs1,^9^ and circZBTB44^10^ have been shown to be expressed differentially in CNV. circ-0007,962 was identified as a differential gene in CNV mice,^11^ and it was shown through the public database Circbank that mmu-circ-0007,962 is a homologue of has-circ-MALAT1. There is, however, no clear mechanism for the action of circ-MALAT1 in CNV. circ-MALAT1 was characterized in laser-induced CNV model mice and Cobalt Chloride (CoCl2-)-induced hypoxic Human Retinal Microvascular Endothelial Cells (HRMECs), and its associated mechanism in CNV was investigated, aiming to the development of its promising potential for the treatment of neovascularization-related diseases.

## Materials and methods

### Ethics statement

All animal protocols were approved by the Guangdong ProvincialAnimal Experiment Center. All mice were handled under the guidelines of the Association for European Vision and Eye Research. Clinical specimens were handled under the Declaration of Helsinki. All patient sgave informed consent before enrollment. This study follows the ARRIVE guidelines.

### Laser-induced CNV model

C57BL/6 J male mice (6‒8 weeks-old) were purchased from Beijing Vital River Laboratory Animal Technology Co., Ltd. (Beijing, China). Laser photocoagulation was performed as described previously.^12^ Briefly, anesthesia was performed by intraperitoneally injecting 1.5 % sodium pentobarbital (100 μL/20 g), and the pupils were dilated with Compound Tropicamide Phenylephrine eye drops, and anesthesia for the ocular surface was administered using 0.4 % oxybuprocaine hydrochloride eye drops. RPE layer was targeted using a covered glass as a contact lens. Utilizing a 532 nm laser (Visulas 532S; Carl Zeiss Meditec, Dublin, Ireland), four evenly distributed laser dots were created around the optic nerve, symmetrically distributed between the primary blood vessels in each eye. Laser injury was induced at 120 mW for 100 ms. Bubbles were seen under the slit lamp, suggesting successful disruption of the Bruch membrane without vitreous hemorrhage.

### Hematoxylin-Eosin staining (HE)

The mouse eyeballs were removed, fixed in 4 % paraformaldehyde for 24 h, dehydrated in 100 %, 95 %, 85 %, and 75 % ethanol for 3 min, paraffin-embedded after xylene treatment, and made into 4 μm paraffin sections, which were then subjected to HE-staining.

### Fluorescein fundus angiography (FFA)

On day 7 after laser modeling, after anesthetizing the mice and surface anesthesia of the eyeballs as previously described, 0.3 mL 2 % fluorescein sodium was injected intraperitoneally, and the FFA images were observed and recorded 2‒3 min after the injection under fluorescence angiography (HRA, Heidelberg, Germany).

### Construction and subretinal administration of AAV2 vectors

The pAAV2-shRNA-circ-MALAT1 and pAAV2-shRNA-Negative Control (NC) were constructed by OBiO Technology (Shanghai, China). Viral titers were diluted to 1 × 10^11^ vg/mL. The procedures for subretinal injections followed previously outlined methods. Anesthetized mice were treated with 0.5 % tropicamide (Ciba Vision Ophthalmics, Blagnac, France) and 0.5 % epinephrine (Altaire Pharmaceuticals, USA) to dilate the pupil. Using a 30 gauge needle, a channel was formed about 1 mm away from the corneal limbus, followed by the immediate injection of 1 μL AAV2 solution into the subretinal area with a 34 gauge needle post-laser photocoagulation. Subretinal drug administration was considered successful if the injection produced a small bubble.

### MiRNA treatment

After anesthesia and pupil dilation, channels were created as previously described,^12^ and 1 μL 50 nM constructs (NC agomir, miR-96–5p agomir, inhibitor NC, miR-96–5p inhibitor; RiboBio, Guangzhou, China) were injected with a 34-gauge needle immediately after laser photocoagulation.

### Cell culture and transfection

HRMECs (ATCC, USA) underwent cultivation in an endothelial cell environment (comprising fetal bovine serum, endothelial cell growth factor, and penicillin/streptomycin) maintained at 37 °C in an atmosphere containing 5 % CO_2_, with passages every three days. Lipofectamine 3000 (Thermo Fisher Scientific) was used to transfect HRMECs. RiboBio manufactured circ-MALAT1 siRNA, FOXK2 overexpression vector, miR-96–5p mimic, and miR-96–5p inhibitor. Cells that underwent transfection were incubated for 48 h before being tested for transfection efficiency.

### RNase R treatment and subcellular localization

In the case of RNase *R* treatment, around 2 μg of total RNA underwent incubation, either with or without 3 U/μg of RNase R, for 30 min at a temperature of 37 °C. The obtained RNA was purified using RNeasy MinElute Cleanup Kit (QIAGEN, USA). CircRNA and MALAT1 mRNA were detected by RT-qPCR.

HRMECs (1 × 10^6^) were treated with PARIS™ Kit (Invitrogen, USA) and centrifuged to separate nuclei and cytoplasm. Gene expression was detected by RT-qPCR. GAPDH was the cytoplasmic reference and U6 was the nuclear reference.

### Cobalt chloride (CoCl_2_) treatment

Transfected or untransfected HRMECs were treated with 150 μM CoCl_2_ for 24 h to establish an endothelial cell hypoxia model.

### CCK-8 assay

HRMECs with completed transfection were inoculated in 96 well plates. After 0 h, 24h , 48h , and 72 h of incubation, CCK-8 reagent (Beyotime) was added at 1:100 for 2 h at 37 °C. The measurement of absorbance was conducted at 450 nm.

### Tube formation assay

The procedures for tube formation assays followed the methods outlined earlier. Portions of Matrigel (150 mL; BD Biosciences) were introduced into 48 well plates and maintained at 37 °C for half an hour. HRMECs were resuspended and inoculated onto the gel (2 × 10^4^ cells/well) and imaged after 8 h in 5 fields. The network of tubular structures was measured using Image Pro Plus software (v.6.0; MediaCybernetic, USA).

### Dual luciferase activity assay

The binding sites between circ-MALAT1 and miR-96–5p, and between miR-96–5p and FOXK2 were predicted in starBase 3.0 (http://starbase.sysu.edu.cn/). GenePharma (Shanghai, China) was commissioned to synthesize miR-96–5p binding site-containing wild-type and mutant circ-MALAT1 fragments, and wild-type and mutant FOXK2, which were cloned into pmirGLO luciferase reporter vector (Promega, USA), respectively, and the resulting plasmids were WT-circ-MALAT1 and MUT-circ-MALAT1, and WT-FOXK2 and MUT-FOXK2, respectively. The plasmids were cotransfected into HRMECs containing miR-96–5p mimic or negative control (mimic-NC) using Lipofectamine® 3000 (Invitrogen). The luciferase activity was measured using the Dual Luciferase Reporter Gene Assay System (Promega) at 48 h and detected with a Synergy 2 Multidetector Microplate Reader (BioTek. USA).

### RNA immunoprecipitation analysis (RIP)

RIP was performed in HRMECs using the Magna RIP™ RNA Binding Protein Immunoprecipitation Kit (Millipore) at 48 h post-transfection. HRMECs were lysed in a complete RNA lysis buffer and incubated with primary antibodies for 3 h at 4 °C (Ago2 or IgG). Immunoprecipitates from bound beads were eluted with an elution buffer. Extracted RNA was analyzed by RT-qPCR.

### Flow cytometry

The process of apoptosis was identified through the Membrane Associated Protein V-FITC kit (KeyGenBiotech, Nanjing, China). Following a 48-hour transfection period, HRMECs underwent a 15-min incubation at ambient temperature in darkness with membrane-anchored protein V-FITC and propidium iodide, after which they were examined using a BD FACSCalibur flow cytometry system (BD Biosciences, USA).

### Caspase 3 activity assay

Caspase 3 was determined by using a Caspase 3 assay kit (Beyotime, Beijing, China). Briefly, HRMECs (1 × 10^6^) were lysed and centrifuged at 10,000 g. Proteins (10 mL) were added to 80 mL reaction buffer and 10 mL Caspase 3 substrate Ac-DEVDpNA (2 mM). After 4 h, Caspase 3 cleavage was monitored using a microplate reader. Monitoring the enzyme-catalyzed release of p-nitroaniline was done by measuring the absorbance at 405 nm.

### RT-qPCR

RNA extraction from HRMECs and RPE choroidal complexes was performed with the Total RNA Extraction Kit (Tiangen, Beijing, China), followed by reverse transcription to cDNA using HiScript III RT SuperMix (Vazyme, China). The quantification of first-strand cDNAs was conducted with the SYBR Green Master Kit (Vazyme, China) on the ViiA 7 Real-Time PCR System (Applied Biosystems), using U6 or GAPDH as a benchmark for the comparative quantification technique. Table 1 displays the sequences of the primers.

**Table 1:** Primer sequences.

	Sequence (5′->3′)
**mmu-circ-0007,962**	F 5’- CTTCACCAAACTGGCTTCG −3’
	R 5’- GGACTTGAGCTGAGGTGCTT −3’
**has-circ-MALAT1**	F 5’- GGCGTTGTGCGTAGAGGAT −3’
	R 5’- TCTTTTCTTCGCCTTCCCGT −3’
	RT:5’-CTCAACTGGTGTCGTGGAGTCGGCAATTCAGTTGAGAGCAAAAA −3’
**mir–96–5p**	F 5’- GCCGAGTTTGGCACTAGCACA −3’
	R 5’- TCAACTGGTGTCGTGGAGTC −3’
**FOXK2 (human)**	F 5’- AAGAACGGGGTATTCGTGGAC −3’
	R 5’- CTCGGGAACCTGAATGTGC −3’
**FOXK2**	F 5’- CCATCAACATTCCAGACACGA −3’
	R 5’- GTTGGCAGCACTGATAGTTCC −3’
**GAPDH (human)**	F 5’- TATGATGATATCAAGAGGGTAGT −3’
	R 5’- TGTATCCAAACTCATTGTCATAC −3’
**GAPDH (mus)**	F 5’- GCCAGCCTCGTCTCATAGACA −3’
	R 5’- GTCCGATACGGCCAAATCC −3’
**U6, (human)**	F 5’- CTCGCTTCGGCAGCACA −3’
	R 5’- AACGCTTCACGAATTTGCGT −3’
**U6, (mus)**	F 5’- GAAAGAAGACGCCGAGAAAGG −3’
	R 5’- GGGAGATGTGGATCTATGTCGT −3’

### Data analysis

G*Power software (G*Power 3.1 statistical software) was used for sample size calculation (α = 0.05, power = 0.8, effect size *d* = 0.9), and a minimum sample size of 16 cases per group was required. Twenty nAMD patients and 21 ARC patients were finally included in this study, which met the minimum sample size requirement. All experimental data were analyzed using GraphPad Prism 8.0 software. Continuous variables were checked for normality using the Shapiro-Wilk test. When normality was established, parametric tests were performed; otherwise, non-parametric tests were selected. For multiple comparisons, the Bartlett test or Levene's test was used to assess the homogeneity of variance, and if the variance was homogeneous, a one-way ANOVA was conducted; otherwise, ANOVA with Welch correction was applied. For comparison of two groups, two-sided Student *t*-test was used for normally distributed data; for comparison of three or more groups, one-way ANOVA was used for normally distributed and homogeneous data, Kruskal-Wallis test was used for non-normally distributed or homogeneous data, and Mann-Whitney *U test* (for two groups) or Kruskal-Wallis test (for multiple groups) was used for non-parametric data, respectively. For multiple comparison correction, Tukey's method was used for ANOVA post-hoc test, Dunn-Bonferroni's method was used for non-parametric multiple comparisons, and Benjamini-Hochberg's method was used to control the false discovery rate for multiple independent tests of the same data set. In terms of confounding factor control, the number of cell passages and serum batches in cellular experiments, as well as the age and gender of mice in animal models, were uniformly controlled at the experimental design stage. All corrected p-values were labeled in the graphs; p less than 0.05 was statistically significant.

## Results

### Circ-MALAT1 is upregulated in laser-induced CNV lesions and hypoxic HRMECs

**Fig 1:**
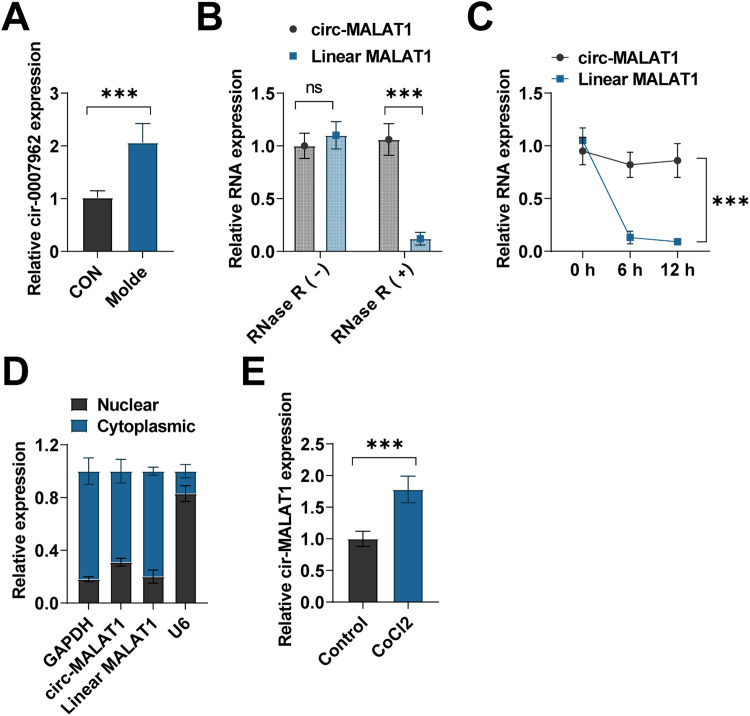
**circ-MALAT1 expression pattern in a laser-induced CNV mouse model.** (A) Expression of circ-0007,962 in CNV mouse model was detected using RT-qPCR (*n* = 5). (B) Total RNA from HRMECs was treated with RNase R. Expression of circRNA and linear RNA was detected by RT-qPCR (*n* = 3). (C) HRMECs were consecutively co-incubated with Actinomycin D. circ-MALAT1 and linear MALAT1 were detected by RT-qPCR (*n* = 3). (D) circ-MALAT1 expression in the nucleus and cytoplasm was detected via nucleoplasmic separation assay and RT-qPCR (*n* = 3). (E) circ-MALAT1 was detected using RT-qPCR in HRMECs treated with CoCl_2_ (*n* = 3). ns, no statistical difference, ***p < 0.001.

The CNY mouse model was induced by laser irradiation. Seven days after laser induction, RT-qPCR was performed on the RPE choroidal complex. has-circ-MALAT1 is a homolog of mouse circ-0007,962. circ-0007,962 relative expression was up-regulated in CNV model mice (Fig. 1A). The stability of circ-MALAT1 was evaluated by exposing HRMECs to RNase R therapy. The RT-qPCR findings indicated that circ-MALAT1, in contrast to linear MALAT1, exhibited resistance to RNase R treatment (Fig. 1B). RNA transcription was inhibited using actinomycin D, and further analysis revealed that circRNA-MALAT1 remained unchanged at its initial state, while the linear degradation of MALAT1 commenced post 6 h of treatment (Fig. 1C). Nucleoplasmic separation analysis showed that circ-MALAT1 was found in both the nucleus and cytoplasm, yet it was notably more abundant in the cytoplasm of HRMECs (Fig. 1D). Hypoxia is recognized as a key driver of CNV formation. HRMECs were exposed to CoCl_2_ to simulate hypoxic conditions, resulting in circ-MALAT1 overexpression (Fig. 1E).

### circ-MALAT1 deficiency inhibits CNV formation in mice

**Fig 2:**
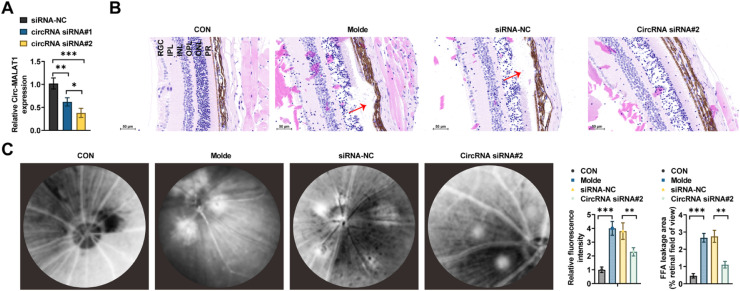
**circ-MALAT1 regulates CNV development *in vivo.*** (A) RT-qPCR verified the knockdown efficiency of circ-0007,962 (*n* = 5). (B) HE-staining was used to observe the structure and integrity of the RPE choroidal complex in the control group and the model mice at 7 days after photocoagulation (50 μm scale). RGC, Retinal Ganglion Cell Layer; IPL, Inner Plexiform Layer; INL, Inner Nuclear Layer; OPL, Outer Plexiform Layer; ONL, Outer Nuclear Layer; PR: Photoreceptor). The arrow points to the location of neovascularization. (C) After successful laser photocoagulation, adenoviral vectors carrying circ-0007,962 control and siRNA for circ-0007,962 were injected subretinally to observe the lesion area fluorescence intensity by FFA assay on day-7 after injury (*n* = 5). ** *p* < 0.01, *** *p* < 0.001.

siRNA oligomers of circ-0007,962 were injected subretinally immediately after CNV induction. RT-qPCR confirmed the knockdown defects, and the most efficient siRNAs were selected for further study (Fig. 2A). RPE choroidal complexes were isolated for CNV examination using HE-staining (Fig. 2B). After 7 days of photocoagulation, the choroidal structure was disorganized and proliferated, with increased angiogenesis in CNV mice; choroidal lesions were reduced in CNV mice after circ-0007,962 deficiency (Fig. 2B). The fluorescence leakage in the choroidal lesion area was then observed by FFA. Control mice showed a papilla-centered radial distribution of retinal blood vessels in the superficial retinal nerve fiber layer, and a reticular distribution of retinal blood vessels in the deeper retinal layer, with intact vascular walls and no obvious fluorescence leakage. CNV mice showed obvious leakage in the fundus with a blurred border, whereas circ-0007,962 knockdown significantly reduced the fluorescence leakage (Fig. 2C).

### circ-MALAT1 deficiency inhibits HRMEC function

**Fig 3:**
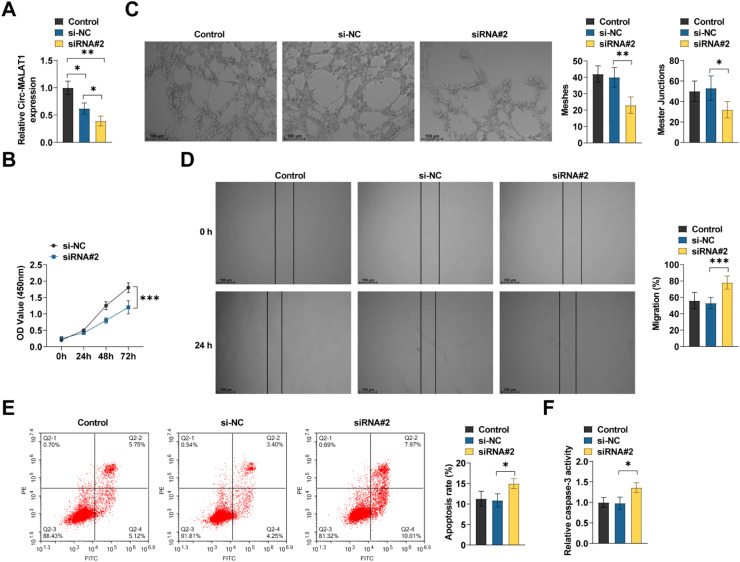
**circ-MALAT1 regulates endothelial function in HRMECs.** (A) siRNAs of circ-MALAT1 were transfected into HRMECs and RT-qPCR was performed to determine knockdown efficiency (*n* = 3). (B) Cell viability was analyzed using CCK-8 assay (*n* = 3). (C) Tube formation assay was performed on matrigel substrates containing HRMECs (*n* = 3). (D) Scratch test was performed to determine the migratory capacity of HRMECs (*n* = 3). (E) Percentage of apoptotic cells was determined by membrane-bound protein V-FITC/propidium iodide staining and flow cytometry (*n* = 3). (F) Relative caspase-3 activity was determined with the caspase-3 activity assay kit (*n* = 3). * *p* < 0.05, ** *p* < 0.01, *** *p* < 0.001.

The authors then tested the effect of circ-MALAT1 silencing on HRMECs under hypoxic stress. siRNA pairs were engineered and introduced into HRMECs to diminish circ-MALAT1 expression. The siRNA knockdown efficacy was assessed using RT-qPCR, leading to the selection of siRNA#2 with superior knockdown efficiency for future research (Fig. 3A). After circ-MALAT1 knockdown, tubule formation was attenuated (Fig. 3C). circ-MALAT1 downregulation inhibited cell activity, reduced cell migration, and increased apoptosis (Fig. 3B, D‒F).

### circ-MALAT1 adsorbs miR-96–5p in HRMECs

**Fig 4:**
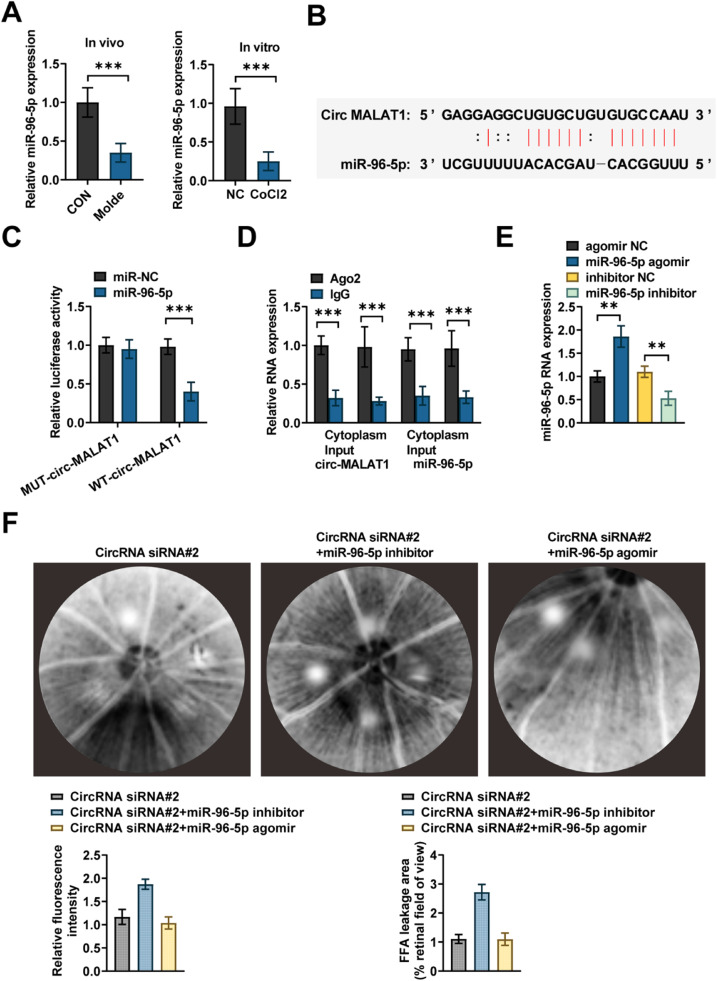
**circ-MALAT1 acts as a miRNA sponge in regulating endothelial function.** (A) miR-96–5p expression was determined by RT-qPCR in ERPE choroidal complex and CoCl_2_-treated HRMECs (*n* = 5). (B) Sequence of potential binding sites for miR-96–5p on circ-MALAT1. (C) HRMECs were cotransfected with circ-MALAT1 wild type or mutants with miR-96–5p mimic or NC. Luciferase activity was assayed (*n* = 3). (D) Cytoplasmic and total cellular fractions were isolated from HRMECs and immunoprecipitated using Ago2 or IgG antibodies. The amount of circ-MALAT1 and miR-96–5p in the immunoprecipitates was determined by RT-qPCR (*n* = 3). (E) RT-qPCR was used to verify overexpression or knockdown efficiency (*n* = 5). (F) FFA assays were performed to observe the lesion area and analyze the fluorescence intensity (*n* = 5). ** *p* < 0.01, *** *p* < 0.001.

Circ-MALAT1 is primarily distributed in the cytoplasm, so the authors believe it functions as a sponge for miRNAs. miR-96–5p was downregulated in CNV mice (Fig. 4A). miR-96–5p level was downregulated in HRMECs with CoCl_2_-induced oxidative stress (Fig. 4A). The presence of potential binding sites for circ-MALAT1 with miR-96–5p was predicted by the Starbase website (Fig. 4B). MiR-96–5p mimic was cotransfected with luciferase reporter constructs mutated in the binding sequence. In the group co-transfected with WT-MALAT1 and miR-96–5p mimic, the luciferase activity was reduced. The mutant MALAT1 eliminated the sponge effect, leaving the activity unchanged (Fig. 4C). RIP analysis revealed that circ-MALAT1 was enriched and miR-96–5p was expressed at a significantly higher level in Ago2-containing immunoprecipitates (Fig. 4D).

### circ-MALAT1/miR-96–5p regulates CNV development

miR-96–5p mimic and inhibitor were designed to upregulate or downregulate miR-96–5p expression, respectively. Following intravitreal injection, miR-96–5p expression in the RPE choroidal complex increased with mimic transfection and decreased with inhibitor transfection (Fig. 4E). The downregulation of miR-96–5p reversed the anti-angiogenesis induced by circ-MALAT1 knockdown. Fundus neovascularization was prone to fluorescence leakage due to its high permeability, resulting in increased fluorescence intensity. miR-96–5p overexpression partially restored this anti-angiogenic formation (Fig. 4F).

**Fig 5:**
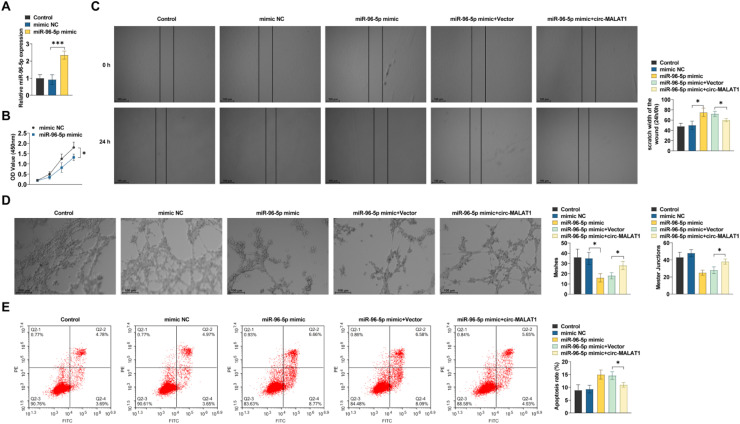
**circ-MALAT1 regulates endothelial function through miR-96–5p** (A) RT-qPCR to verify overexpression efficiency (*n* = 3, * *p* < 0.05). (B) Cell viability was analyzed using CCK-8 assay (*n* = 3). (C) Scratch test was performed to assess the migratory ability of HRMECs (*n* = 3). (D) Tube formation assay was performed to assess tube formation ability (*n* = 3). (E) Flow cytometry was conducted to determine apoptosis (*n* = 3). * *p* < 0.05, ** *p* < 0.01, *** *p* < 0.001.

HRMECs induced by CoCl_2_ were transfected with miR-96–5p mimic or control. miR-96–5p was verified by RT-qPCR (Fig. 5A). miR-96–5p overexpression impeded HRMEC viability, tubule formation, and migration, and promoted apoptosis (Fig. 5B‒E). miR-96–5p elevation had a similar effect to circ-MALAT1 knockdown, both of which exhibit inhibition of endothelial cell progression. Whereas circ-MALAT1 overexpression rescued the endothelial cell proliferation, migration, and tubule formation and promoted apoptosis inhibited by miR-96–5p mimic (Fig. 5B‒E).

### Circ-MALAT1-miR-96–5p-FOXK2 regulates endothelial angiogenesis

**Fig 6:**
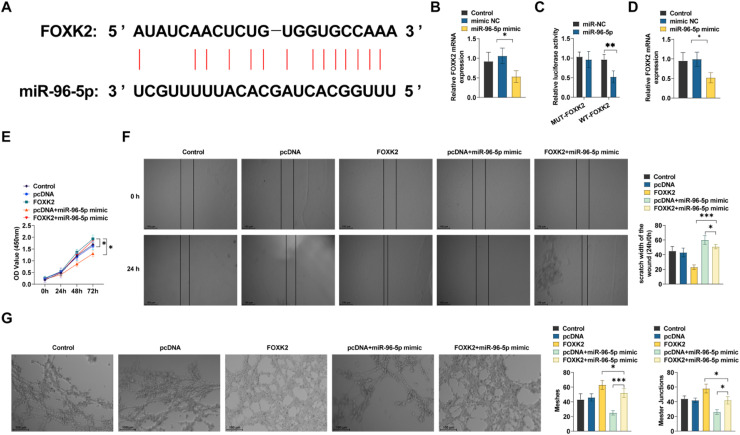
**miR-96–5p/FOXK2 network is involved in regulating CNV development.** (A) Predicted binding sites of miR-96–5p and FOXK2 in public datasets. (B) RT-qPCR was performed to detect FOXK2 expression (*n* = 3). (C) FOXK2 wild type or mutant and miR-96–5p mimics were co-transfected into HRMECs. FLuciferase activity was detected (*n* = 3). (D) RT-qPCR was performed to detect FOXK2 expression (*n* = 3). (E) Cell viability was analyzed using CCK-8 assay (*n* = 3). (F) Scratch test was performed to assess the migratory ability of HRMECs (*n* = 3). (G) Tube formation assay was performed to assess tube formation ability. * *p* < 0.05, *** *p* < 0.001.

Using the Targetscan database to predict the target genes of miR-96–5p, the FOXK2 candidate gene was identified, and the binding site was predicted (Fig. 6A), which has been reported to positively regulate the VEGF and VEGFR signaling networks by FOXK2.^13^ miR-96–5p mimic transfection significantly inhibited FOXK2 in HRMECs (Fig. 6B). The miR-96–5p-FOXK2 targeting correlation was confirmed using a dual luciferase reporter gene assay. The introduction of miR-96–5p mimic reduced the luciferase function in the reporter structure that contained the FOXK2 target sequence (Fig. 6C). miR-96–5p overexpression inhibited FOXK2 expression (Fig. 6D). Further functional analysis showed that overexpressing FOXK2 resulted in a significant enhancement of cell viability, migration, and tubule formation in HRMECs (Fig. 6E‒G); whereas miR-96–5p mimic partially inhibited the pro-endothelial cell progression phenomenon induced by FOXK2 overexpression (Fig. 6E‒G).

**Fig 7:**
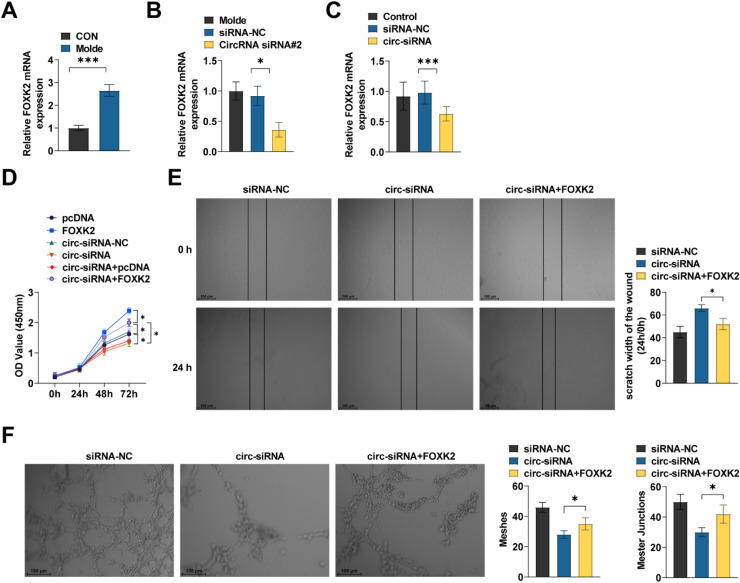
**circ-MALAT1/miR-96–5p/FOXK2 network is involved in regulating CNV development.** (A) RT-qPCR was performed to detect FOXK2 expression in the RPE choroidal complex of mice and CNV mice (*n* = 5). (B) CNV mice received intravitreal injection of CircRNA blank plasmid (siRNA-NC) and circ-MALAT1 knockdown plasmid (CircRNA siRNA#2). On the 7th day after laser treatment, RT-qPCR was performed to detect the expression of FOXK2 in the PRE choroidal complex (*n* = 5). (C) RT-qPCR was performed to detect FOXK2 expression (*n* = 3). (D) Cell viability was analyzed using CCK-8 assay (*n* = 3). (E) Scratch test was performed to assess the migratory ability of HRMECs (*n* = 3). (F) Tube formation assay was performed to assess tube formation ability (*n* = 3). * *p* < 0.05, *** *p* < 0.001.

In laser-induced CNV, FOXK2 mRNA expression was significantly upregulated (Fig. 7A). circ-MALAT1 silencing reduced FOXK2 expression in the choroid (Fig. 7B). In HRMECs with CoCl_2_-induced hypoxia, circ-MALAT1 silencing reduced FOXK2 mRNA expression (Fig. 7C). FOXK2 overexpression partially rescued the action of circ-MALAT1 silencing on HRMECs (Fig. 7D‒F).

### circ-MALAT1 is clinically significant in patients with CNV

**Fig 8:**
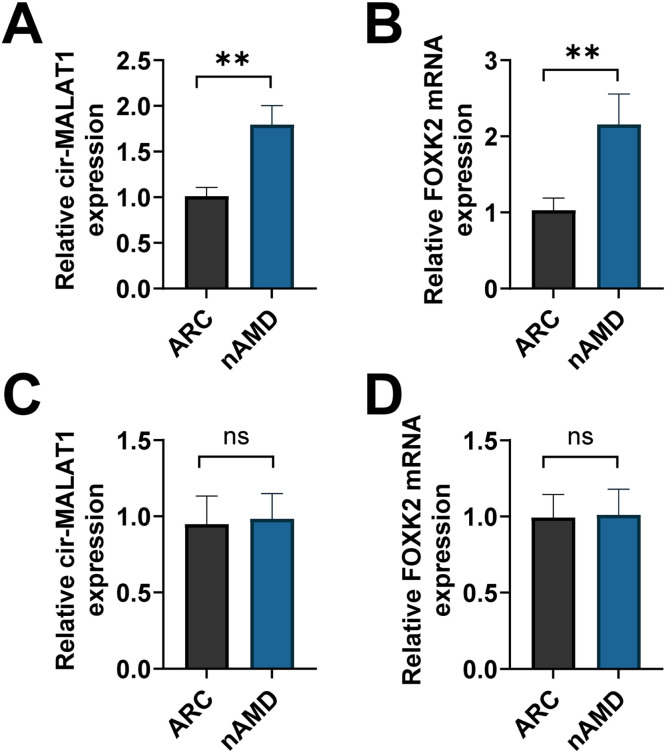
**circ-MALAT1 clinical relevance in CNV patients.** (A‒D) RT-qPCR detected circ-MALAT1 and FOXK2 in AH and plasma of nAMD (*n* = 20) and ARC (*n* = 21) patients. ns, no statistical difference, ** *p* < 0.01 (Age and gender were adjusted as confounding factors, and the results were corrected by Benjamin Hochberg).

The hallmark of nAMD is CNV. To apply our results in a context pertinent to physiology, RT-qPCR was conducted to analyze circ-MALAT1 in the Aqueous Humor (AH) and plasma segments of nAMD patients. In patients with nAMD, there was a notable increase in circ-MALAT1 and FOXK2 levels in the AH, unlike in individuals with age-related cataract (ARC) (Fig. 8A‒B). However, the authors did not find significant differences in plasma circ-MALAT1 and FOXK2 in the 2 cohorts of patients (Fig. 8C‒D). Although circ-MALAT1 and FOXK2 expression in AH was significantly higher in nAMD patients than in the ARC group (*p* < 0.05, corrected for gender-age), this result needs to be further validated in a larger cohort due to the limited sample size (*n* = 20 vs. 21).

## Discussion

Human diseases are affected by circRNA dysregulation, and this study focused on the exploration of CNV-related mechanisms, revealing for the first time the regulatory role of the circ-MALAT1/miR-96–5p/FOXK2 axis in CNV. On the one hand, it was experimentally demonstrated that circ-MALAT1 was upregulated in laser-induced CNV mice and CoCl₂-induced HRMECs, and that circ-MALAT1 enhances FOXK2 expression via miR-96–5p On the other hand, it was first demonstrated that circ-MALAT1 and FOXK2 are highly expressed in AH of nAMD patients relative to ARC patients, and it was also found that the area of fluorescein leakage induced by laser-induced injury was reduced after silencing of circ-MALAT1, and the angiogenic process was inhibited. It should be emphasized, however, that these findings are preliminary evidence, especially limited by the clinical sample size (*n* = 20 vs. 21), and the circ-MALAT1/miR-96–5p/FOXK2 network may still be a therapeutic target for CNV.

CircRNAs are present in a wide range of cells in eukaryotes and are dysregulated in many human diseases.^14^ CircRNAs have been reported to have multiple functions. For example, in diabetic cataract, circRNA KMT2E is up-regulated in the lens and regulates the lens by acting as a sponge with miR-204–5p.^15^ Down-regulating circRNA ZNF532 regulates ocular-associated disease pathology through different miRNAs^.^^16^ CircRNA HIPK3 acts as an endogenous miR-30a-3p sponge, mediating diabetic retinal vascular dysfunction.^17^ circRNA RSU1 mediates miR-345–3p, which mediates inflammatory responses and oxidative stress, leading to vascular dysfunction.^18^ CNV pathology also involves endothelial cell proliferation, migration, and angiogenesis, all of which are mediated by circRNAs.^19^

CNV represents a category of pathological angiogenesis in the eye. Angiogenesis development encompasses the stimulation of vascular endothelial cells, breakdown of the extracellular matrix, migration and proliferation of endothelial cells, tight junction formation, engagement of pericytes, and the accumulation of a new basement membrane.^20^ Several studies have demonstrated the involvement of non-coding RNAs in the regulation of AMD.^7^^,^^21^ VEGF is known to be involved in vascular alterations, such as AMD,^22^ diabetic retinopathy^23^ and retinal vein occlusion.^24^ For example, circ-MALAT1 has been detected to be significantly upregulated in a CVN mouse model.^11^ Similar results were found in the present study, where circ-MALAT1 was highly expressed in the AH of CNV model mice and nAMD patients. The specific expression of circ-MALAT1 was knocked down by subretinal injection of AVV2 vector carrying siRNA, and the downregulation of circ-MALAT1 attenuated the structural disorganization of the choroid caused by laser photocoagulation and was marked by hyperplasia, increased blood vessel formation, and fluorescence leakage from the fundus (Fig. 2B‒C). By extracting nuclear/cytoplasmic RNA and detecting circ-MALAT1 expression, the authors determined that circ-MALAT1 is predominantly distributed in the cytoplasm of HRMECs, and they further hypothesized that it functions through miRNA uptake. Through the public database StarBase database, miR-96–5p was identified as a potential target. miR-96–5p has a potential binding site for circ-MALAT1 with a predicted TargetSite of chr11:65,503,077–65,503,100 [+]. miR-96–5p can regulate cell proliferation and migration in various diseases, such as hepatocellular carcinoma^25^ and ovarian cancer.^26^ miR-96–5p was lowly expressed in a CNV mouse model and HRMECs induced by hypoxia. Intravitreal injection of miR-96–5p agomir reduced the size of neovascularized areas, and overexpressing miR-96–5p decreased the proliferation and migration of HRMECs. Intravitreal injection of miR-96–5p inhibitor reversed circ-MALAT1 knockdown-induced antivascularization and reduced fundus fluorescence leakage. circ-MALAT1 overexpression partially abolished the action of miR-96–5p mimic on cell proliferation, migration, and tubule formation. It was further demonstrated that circ-MALAT1 affects CNV through miR-96–5p regulation.

Currently, it is recognized that hypoxia theory, local tissue hypoxia, and inflammatory response are likely to cause hypoxia in the retina outside the macula, which produces angiogenic factors to stimulate choroidal capillary neovascularization, forming subretinal neovascularization.^27^ CoCl_2_ is widely used as a hypoxia agent for *in vitro* modeling of chemically induced hypoxia by stabilizing HIF-1α protein expression.^28^ The authors used CoCl_2_-induced hypoxia in HRMECs to mimic an *in vitro* hypoxia model. miR-96–5p overexpression was followed by decreased expression of FOXK2 (Fig. 6B), a member of the FOX family of transcription factors that have been reported to be required for cell proliferation and survival.^29^ In a thyroid undifferentiated carcinoma, FOXK2 promotes angiogenesis by inducing VEGFA transcription.^13^ Vascular leakage and inflammation caused by excessive VEGFA release are known to play a key role in CNV and exudative AMD.^30^ FOXK2 was confirmed as a potential target of miR-96–5p by public databases (Fig. 6A). Further *in vitro* functional analyses showed that FOXK2 overexpression partially rescued the effect of circ-MALAT1 silencing on hypoxic HRMECs. It is important to emphasize that although CoCl_2_ can effectively mimic acute hypoxic stress by stabilizing HIF-1α protein, its induced hypoxic environment lacks the synergistic effects of chronic inflammation (*e.g.*, complement activation, macrophage infiltration) and metabolic imbalance (*e.g.*, lipid peroxidation)^31^ in the retina of nAMD patients. This simplified modeling may limit the direct extrapolation of findings to complex pathological microenvironments. In this regard, a mouse model of laser-induced CNV was designed. The results showed that the choroidal structure of CNV mice was disorganized, accompanied by increased angiogenesis, similar to a previous study showing that choroidal lesions in CNV mice are significantly reduced after circ-0007,962 deficiency.^32^ Mechanistically, in CNV mice, circ-MALAT1 knockdown significantly inhibited CNV progression (Fig. 4F) and decreased relative FOXK2 mRNA expression (Fig. 7B). On functional validation, miR-96–5p overexpression partially reversed the inhibitory effect of circ-MALAT1 knockdown on CNV (Fig. 4F). This was demonstrated by the downregulation of miR-96–5p reversing the anti-angiogenesis induced by circ-MALAT1 knockdown, which led to neovascularization resulting in increased fluorescence intensity. This is due to the fact that fundus neovascularization is prone to fluorescence leakage due to its high permeability, leading to increased fluorescence intensity.^33^ At the same time, miR-96–5p overexpression restored this antiangiogenesis, decreasing the fluorescence leakage and attenuating the fluorescence intensity. These results have supported a direct test of the effect of miR-96–5p on CNV progression in mice. However, the causality was not verified in this study by direct manipulation of miR-96–5p or FOXK2 *in vivo* models (*e.g.*, endothelial cell-specific FOXK2 knockdown or AAV-mediated miR-96–5p overexpression). Further confirmation of whether this axis directly drives CNV processes in gene-edited animal models is needed in the future. In terms of mechanistic validation, the functional necessity was confirmed by siRNA-specific knockdown of circ-MALAT1 (Fig. 2A) and miR-96–5p mimic/inhibitor (Fig. 4E‒F). It should be noted, however, that siRNAs may non-specifically interfere with linear MALAT1 or other cognate RNA molecules. Although actinomycin D experiments (Fig. 1C) and RNase R treatment (Fig. 1B) demonstrated the independent stability of circ-MALAT1 from linear transcripts and RT-qPCR showed no significant change in linear MALAT1 expression, it is still necessary to further validate target specificity by the following strategy: 1) CRISPR/Cas9 gene editing targeting the circ-MALAT1 reverse splice site; and 2) Single-cell transcriptome sequencing to comprehensively assess gene expression profile bias. In addition, miR-96–5p overexpression may affect other signaling pathways through off-target effects, which need to be combined with RIP-seq and proteomics to screen potential off-target molecules. Finally, on the corroboration of pathological correlations in clinical samples, the results confirmed that circ-MALAT1 was synchronously upregulated with FOXK2 in the AH of patients with nAMD (Fig. 8A‒B), suggesting the potential pathological significance of this axis in human CNV and indirectly supporting its value as a therapeutic target.

Anti-VEGF therapies (*e.g.*, ranibizumab, abciximab) are still the first-line treatments for nAMD, but their efficacy is limited by patient resistance and invasive maneuvers of repeated vitreous injections.^34^ The therapeutic strategy of targeting the circ-MALAT1/miR-96–5p/FOXK2 axis proposed in this study may provide a new direction of intervention for nAMD: on the one hand, this mechanism has the potential to be mechanistically complementary to the VEGF/VEGFR signaling pathway. Unlike anti-VEGF, which directly neutralizes VEGFA, circ-MALAT1 silencing inhibited FOXK2 through upregulation of miR-96–5p (Fig. 7B‒C), which suppressed HRMEC viability, migration, and angiogenesis and promoted apoptosis. It is known that endothelial cell viability (proliferation and survival capacity) is the basis of angiogenesis.^35^ Endothelial cell migration is a necessary step in angiogenesis.^36^ Driven by pro-vascular factors such as VEGF, HRMECs migrate directionally towards hypoxic regions through integrin-mediated cytoskeletal reorganization and matrix metalloproteinase secretion.^37^ In addition, tube formation is a hallmark of endothelial cell differentiation and vascular lumen formation.^37^ It was hypothesized that this upstream regulation may delay the development of VEGF resistance and provide a theoretical basis for combination therapy. On the other hand, in a mouse model, circ-MALAT1 knockdown reduced the fluorescence leakage area (Fig. 2C), suggesting its therapeutic potential in inhibiting neovascularization. In a study of AMD, combined inhibition of prostaglandins and VEGF is effective in reducing vascular leakage and neovascularization, showing better efficacy than single inhibition of VEGF. Although this study did not directly compare or combine anti-VEGF monotherapy, miR-96 family members have been observed to affect the blood-retinal barrier by regulating VEGFR2 in diabetic retinopathy,^38^ and a retinal vein occlusion model showed that hypoxia-induced circRNA abnormalities activate pro-angiogenic pathways. In diabetic retinopathy, FOXK2 has been shown to promote retinal endothelial cell migration by regulating reprogramming of glucose metabolism.^39^ This evidence echoes the molecular mechanism of the present study, suggesting that targeting this regulatory axis may have synergistic inhibitory effects on a variety of fundus neovascularization diseases. In particular, in cases where VEGF treatment resistance exists (*e.g.*, resistance due to VEGFR2 mutations in diabetic retinopathy), intervening in the circ-MALAT1/miR-96–5p/FOXK2 network may provide an alternative therapeutic strategy.

This study has the following limitations. Smaller clinical sample sizes (*n* = 20 nAMD *vs.* 21 ARC) may increase the risk of type II error and preclude subgroup analyses (*e.g.*, anti-VEGF treatment responders *vs.* non-responders). Invasive collection of AH samples limits the rapid expansion of large-scale cohorts. CoCl₂-induced hypoxia model *in vitro* fails to fully mimic the chronic inflammatory microenvironment of the retina in nAMD patients. Lack of direct *in vivo* functional validation (*e.g.*, endothelial-specific knockout or overexpression models) of miR-96–5p and FOXK2 leads to evidence of causality in the regulatory axis that still needs to be refined. In addition, endothelial cell-specific FOXK2 knockout mice need to be constructed to observe the changes in their sensitivity to laser-induced CNV. Subretinal delivery of miR-96–5p mimics by AAV vectors is required to assess their efficacy in monotherapy or combination with anti-VEGF therapy. In addition, it is necessary to expand the sample size through multicenter collaboration and verify the generalizability of this study in combination with patient primary cell and organoid models. In conclusion, the present study preliminarily suggests that the circ-MALAT1/miR-96–5p/FOXK2 axis may be involved in the pathological progression of CNV by regulating the VEGF signaling pathway. Although its direct causal role in CNV needs to be further validated by endothelial-specific gene manipulation modeling, the mechanistic insights of this study provide a basis for the development of new combination therapies or alternative strategies, suggesting that the regulatory mechanism of this axis may provide new directions for CNV therapy, although its clinical translational value has not been fully demonstrated.

## Availability of data and materials

The datasets used and/or analyzed during the present study are available from the corresponding author on reasonable request.

## Ethics approval

All animal protocols were approved by the Guangdong Provincial Animal Experiment Center (n° CJ2023–248). All mice were handled under the guidelines of the Association for European Vision and Eye Research.

And all procedures performed in this study involving human participants were in accordance with the ethical standards of the institutional and/or national research committee and with the 1964 Helsinki Declaration and its later amendments or comparable ethical standards. All subjects were approved by the China-Japan Friendship Hospital (2023-KY-274). All patients gave informed consent before enrollment.

## Funding

National High Level Hospital Clinical Research Funding (2023-NHLHCRF-YYPPLC-ZR-21).

## CRediT authorship contribution statement

**Yue YIN:** Formal analysis, Funding acquisition, Project administration, Software, Supervision, Visualization, Writing – original draft. **Tong Zhao:** Conceptualization, Data curation, Investigation, Methodology, Resources, Validation, Writing – review & editing.

## Declaration of competing interest

The authors declare no conflicts of interest.
